# Clinical Efficacy of CBCT and 3D‐Printed Replicas in Molar Autotransplantation: A Controlled Clinical Trial

**DOI:** 10.1111/edt.13012

**Published:** 2024-11-06

**Authors:** Miks Lejnieks, Ilze Akota, Gundega Jākobsone, Laura Neimane, Sergio E. Uribe

**Affiliations:** ^1^ Department of Oral and Maxillofacial Surgery and Oral Medicine Riga Stradins University Riga Latvia; ^2^ Baltic Biomaterials Centre of Excellence Headquarters at Riga Technical University Riga Latvia; ^3^ Institute of Stomatology Riga Stradins University Riga Latvia; ^4^ Institute of Stomatology Implantology Clinic Riga Stradins University Riga Latvia; ^5^ Department of Orthodontics Riga Stradins University Riga Latvia; ^6^ Department of Conservative Dentistry and Oral Health Riga Stradins University Riga Latvia; ^7^ Department of Conservative Dentistry and Periodontology, LMU University Hospital LMU Munich Munich Germany

**Keywords:** cone beam computed tomography, immature, printed tooth replica, third molar, tooth transplantation

## Abstract

**Background/Aim:**

To evaluate the efficacy of the combined cone‐beam (CBCT)/3D‐replicas protocol on the clinical and radiographic outcomes of autotransplanted molars.

**Material and Methods:**

Controlled clinical trial registered ISRCTN13563091 from August 2019 to September 2022. Patients aged 13–22 years requiring permanent premolar extraction and having at least one non‐erupted third molar were enrolled at the Institute of Stomatology, Stradins University, Riga, Latvia. Patients in the 3D‐replicas (*n* = 30) underwent maxillary CBCT scans and had 3D‐printed replicas of the third molar fabricated, while the control group (*n* = 28) did not. The clinical outcomes included tooth mobility, bleeding on probing, and periodontal pocket depth assessed at 3, 6, and 12 months. The radiographic outcomes included root development, obliteration, periapical status, and crown changes at 12 months.

**Results:**

Of the 55 patients assigned to interventions, 46 completed the study. No significant differences in survival and radiographic outcomes were found between the control (*n* = 22) and 3D‐replica group (*n* = 24): root development (*p* = 0.3), root resorption (*p* = 0.057), periapical status (*p* = 0.7), and crown/root ratio change (*p* = 0.4). Logistic regression showed no significant associations between radiologic predictors (root resorption: *p* = 0.4; periapical status: *p* > 0.9; root development: *p* = 0.8). Significant clinical outcome predictors included total operative time (*β* = 0.0043, *p* = 0.049), Moorrees' stage (stage 4: *β* = −0.31, *p* < 0.001; stage 5: *β* = −0.39, *p* < 0.001), and four donor placement times (*β* = 0.93, *p* < 0.001), but group assignment was not a significant predictor.

**Conclusions:**

The CBCT/3D‐replica protocol showed no significant differences in the clinical or radiological outcomes. The high success rates in both groups suggest that the protocol is valuable primarily for optimizing surgical efficiency and as a training tool for clinicians.

## 1 Introduction

Tooth autotransplantation is a biological treatment for missing teeth. It is an alternative to dental implants [1] to replace teeth damaged by caries or trauma [2]. This effectively addressed the challenge of bone growth in younger patients [3, 4]. Long‐term studies reported a survival rate of 97.9%, with 1‐year survival at 98% and 5‐ and 10‐year rates at 95.9% and 96.9%, respectively [5]. Survival rates are 95.6% for children and adolescents and 80.3% for adults [2, 5, 6].

With the introduction of cone beam computed tomography (CBCT) [7] and three‐dimensional printers [8], three‐dimensional replicas can be created for enhanced dental procedure accuracy and efficiency [9]. These technological advances have been instrumental in improving surgical technical performance [2, 10]. CBCT‐guided tooth autotransplantation in children has shown an 8% higher survival rate and success rate than conventional methods [7]. Additionally, a recent clinical trial with a reproducible protocol demonstrated that combining CBCT and 3D‐printed replicas significantly enhances surgical efficiency in molar autotransplantation by reducing extra alveolar time [9, 11], intraoperative fitting attempts [9], and total surgical time [12], hence potentially reducing the risk of injury to periodontal structures. Recent systematic reviews have reported high success and survival rates for tooth autotransplantation using 3D‐replicas based on observational evidence from multiple prospective and retrospective studies [13]. Despite these advances, most reported outcomes for using CBCT and 3D‐replicas for autotransplantation are derived from observational studies, case reports, and a handful of case–control studies [6, 14, 15]. However, no evidence from controlled clinical trials has been reported [5, 6, 14]. Previously, we reported the technical efficacy of 3D‐replicas for guided autotransplantation in a controlled clinical trial, showing improved surgical efficiency [12]. However, the biological outcomes, particularly the clinical and radiological results at 1‐year follow‐up, have not yet been reported. Therefore, this study aimed to evaluate the efficacy of the combined protocol of CBCT and 3D‐replicas in improving the clinical and radiologic outcomes of autotransplanted molars in a controlled clinical trial.

## 2 Materials and Methods

### 2.1 Trial Design, Report, and Implementation

This non‐randomized controlled clinical trial adhered to the principles of the Declaration of Helsinki. The study was implemented according to the protocol approved by the Ethics Committee of Riga Stradins University (RSU) (approval Nr. 6–1/08/12), available at ISRCTN13563091 (https://doi.org/10.1186/ISRCTN1356309.25). The trial was implemented and reported following CONSORT recommendations (Table S1).

### 2.2 Participants

Between August 2019 and September 2022, 95 patients aged 13–22 who required permanent premolar, first, or second molar extractions and had at least one non‐erupted third molar were referred to RSU's Institute of Stomatology. Of these, 42 were excluded, and 53 were assigned to the intervention. To ensure full understanding and written informed consent, the participants were thoroughly informed of the study objectives, procedures, benefits, and potential risks. The participants were guaranteed their right to withdraw without any consequences.

The inclusion criteria were: clinically healthy and not pregnant patients, aged under 25 years, with at least one premolar or molar absent, designated for replacement by an autotransplanted third molar, understanding of the procedure with documented written consent, presence of unerupted third molars independently of root anatomy and with Moorrees stages 3–5 [16], good oral hygiene (no gingivitis), and non‐smokers. All 46 participants who were invited met the criteria and were included in the study. The exclusion criteria included the inability to attend scheduled control visits or a history of traumatic extraction of the donor's tooth. Due to limited CBCT availability during the COVID‐19 pandemic, patients were sequentially assigned to groups, with priority given to the 3D‐replica group during allocated CBCT slots.

### 2.3 Interventions

Patients in the 3D group underwent a maxillary CBCT scan (i‐CAT Next Generation, Kavo, Germany), tube voltage 120 k, 5 mA, exposure time of 4 s, 0.3 mm voxel size, and 13 × 16 cm field of view. The 3D‐printed replica of the third molar was fabricated according to a previously published protocol [12]. CBCT was not performed on patients in the control group. Each patient underwent a recipient site analysis. All patients had a sufficient vertical height of the mandibular nerve. All procedures were planned and performed by a single experienced surgeon who had performed > 20 autotransplantations before the study. Due to the different procedures, it was impossible to blind the surgeon to the type of procedure. Preoperative antibiotics were administered as either amoxicillin/clavulanic acid or clindamycin in cases of penicillin allergy. The patient did not receive any additional antibiotics. In the recipient region, osteotomy was carried out with a straight fissure and surgical round burs, along with implant burs from Straumann BLT (Basel, Switzerland). Saline from an implanted Kavo Physio dispenser (KaVo, Biberach an der Riss, Germany) was used for cooling, while the drill speed was limited to 800 rpm. After the extracted tooth socket was adapted, the immature and unerupted autotransplanted tooth (root ½ to open apex, Moorrees stages 3–5) was placed into the prepared alveolus or osteotomy and positioned in supragingival infraocclusion. Depending on the surgical needs, suturing was done using either Silk 3‐0 or Vicryl 4‐0.

**FIGURE 1:**
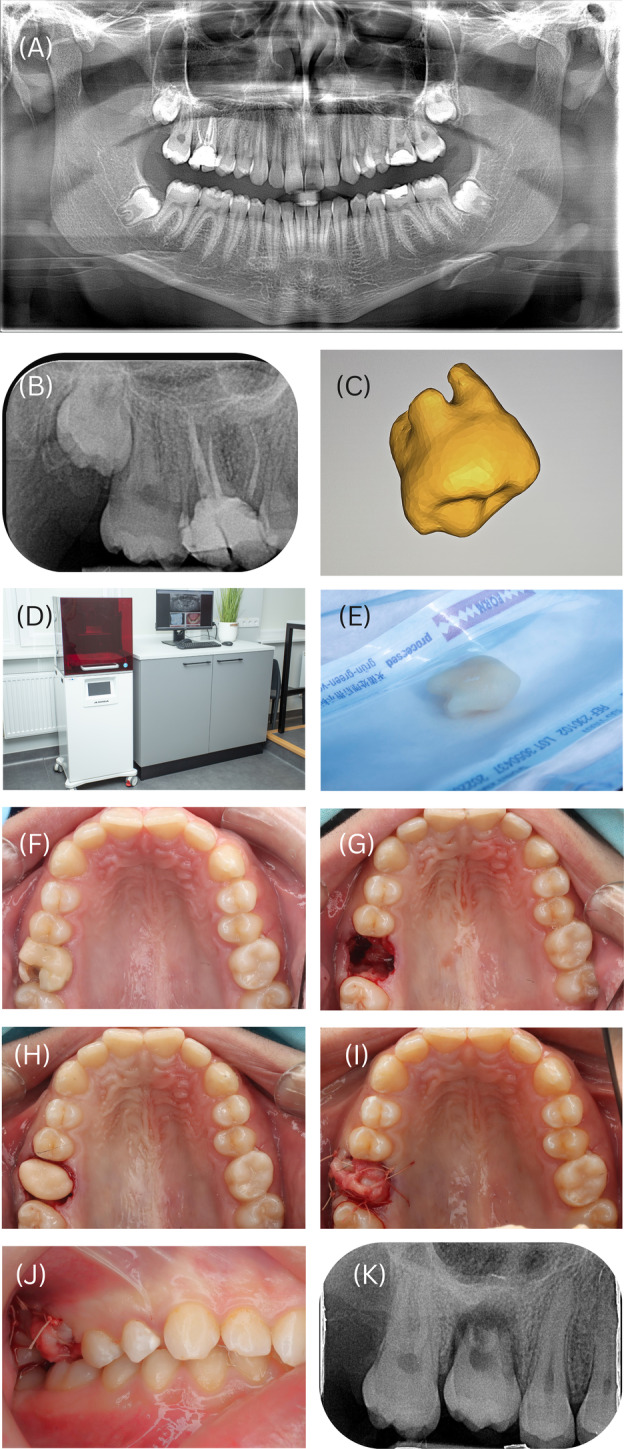
Autotransplanting an immature third molar from d18 to the d16 location. (A) Pre‐treatment orthopantomogram of a 17‐year‐old male. The upper right first molar was replaced for endodontic reasons with an unerupted upper right third molar. The donor tooth had root development at stage 3 [16] and developed 1/2 of the projected root length. (B) A periapical radiograph was obtained 1 month before surgery. (C) The 3D‐printed model was created according to the DOI:10.1111/edt.12905 protocol. (D) Asiga Max UV printer unit (Asiga, Alexandria, NSW, Australia). (E) The replica underwent sterilization at 121°C in a two‐atmosphere steam cycle, taking 60 min to complete. (F) Occlusal view before auto‐transplantation. (G) Saline‐irrigated burs were employed to prepare the recipient site after extraction. (H) Fitting of the 3D‐replica. (I) Sutures are used to stabilize the transplant. (J) Transplanted tooth test in occlusion. (K) A periapical radiograph was taken on the day of surgery.

Control group patients did not receive CBCT scans. Extracted teeth were reimplanted following standard protocols without 3D‐printed replicas [17]. In the 3D group, the tooth replica model was adapted to the recipient site before autotransplantation. If the donor tooth did not fit well, it was placed back in its original socket while adjustments were made to the recipient site. The extra‐alveolar time was recorded for both groups, ending when the donor tooth was placed into the recipient socket. All surgical and 3D printing steps are shown in Figure 1.

### 2.4 Outcomes

#### 2.4.1 Clinical Outcomes

Tooth mobility, bleeding on probing, and periodontal pocket depth [18] were measured in all patients at 3, 6, and 12 months by a single surgeon blinded to patient allocation.

1. Tooth mobility: Tooth mobility was assessed using the blunt ends of the two dental mirrors. Measurements were as follows, 0: normal physiological mobility (0.1–0.2 mm in the horizontal axis); 1: mobility greater than 1 mm in the horizontal plane (buccolingual or mesiodistal).
2. Periodontal pocket depth: The periodontal pocket depth was measured using the manual probing technique with a Williams probe. Each molar was examined in six positions: distofacial, facial, mesiofacial, distolingual, lingual, and mesiolingual. The deepest pocket depth at each position was recorded and categorized as 0: normal (up to 3 mm); 1: periodontal pocket greater than 3 mm.
3. Bleeding on probing: Bleeding on probing was assessed using the Williams probe. Each molar was probed at six positions: distofacial, facial, mesiofacial, distolingual, lingual, and mesiolingual. After probing, the sites were observed for immediate and delayed bleeding (10–15 s after probing). Bleeding was categorized as 0: Normal (no bleeding observed); 1: bleeding observed.

#### 2.4.2 Radiological Outcomes

A researcher prepared a set of 13 radiographs for the radiological evaluation. Two expert radiologists (LN/SU) with over 10 years of experience participated in a calibration session before independently assessing the radiographs at their workstations. Both radiologists were blinded to the patient groups. The measured parameters included root development, obliteration, periapical status, and crown change [18, 19].

1. Root development was assessed by comparing the current radiographs with the baseline. Status was categorized as no change, improved (+), worsened (−), or not available/other.
2. Root resorption was evaluated by noting the presence of any lateral or non‐apical root resorption, which was recorded as no, yes, or not available (NA).
3. Periapical status was categorized as normal (0), widening periodontal space or loss of lamina dura (1), radiolucent periapical lesion (2), NA, or others.
4. The crown/root ratio change was determined by measuring the crown and root dimensions in pixels at baseline and at the final examination to establish the crown/root ratio. Measurements included the crown baseline (measured from the mesial more occlusal point to the cervico‐enamel junction, or NA if no tooth was present), root baseline (measured from the mesial cervico‐enamel junction to the most apical root part, or NA if no tooth was present), crown final (measured similarly to the crown baseline at the final examination), and root final (measured similarly to the root baseline at the final examination).

The agreement statistics for these evaluations were then calculated. Cohen's Kappa for root development was 0.755 (*z* = 2.81, *p* = 0.005), root obliteration was 0.629 (*z* = 2.44, *p* = 0.0146), and periapical status was 0.845 (*z* = 3.79, *p* < 0.01). The intraclass correlation coefficient for the final crown measurement was 1.0, indicating excellent reliability (*F*(12, 13) = Inf, *p* < 0.01). A new calibration session would have been scheduled if any agreement value was below 70%, but this was unnecessary given the excellent agreement for all criteria.

### 2.5 Sample Size Calculation

The sample size for this study was determined based on the primary outcome measure of tooth mobility [20]. Assuming a difference in the proportion of teeth with mobility from ~50% in the control group to 20% in the 3D‐replica group, a clinically significant difference of 30%, the effect size was calculated using Cohen's h. Each group contained approximately 22 participants to achieve 80% power at a significance level of *p* < 0.05.

### 2.6 Statistical Analysis

The radiological outcomes were analyzed to compare the 3D‐replica and Control groups. The Wilcoxon rank‐sum test was used to analyze crown and root changes. Fisher's exact test was used to assess the distribution of the periapical status, root development, and external root resorption. Additionally, a multivariate logistic regression analysis was conducted to evaluate the association between various radiological predictors and the likelihood of belonging to the 3D‐replica or Control group. The logistic regression model included root development, root resorption, periapical status, and crown/ratio changes as the dependent variables. This analysis aimed to adjust for potential confounders. Generalized linear mixed models (GLMMs) were fitted to predict mobility, bleeding, and pocket depth outcomes for clinical outcomes. The models included the group (3D‐replica vs. control), time, age at surgery, sex, total surgery time, extra‐alveolar time, Moorrees' Stage, and donor fitting times as fixed effects, with a random intercept for each patient. The significance level was set at 5%. All statistical analyses were performed using the R software.

## 3 Results

### 3.1 Study Population

**FIGURE 2:**
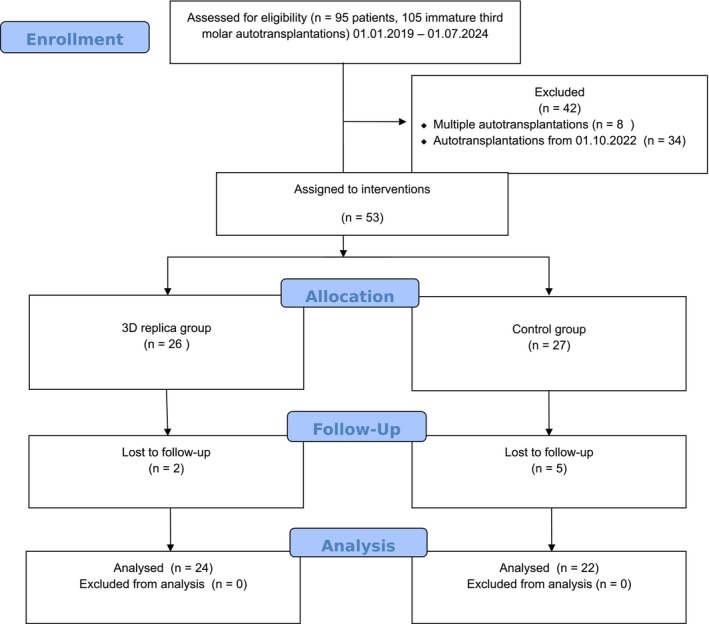
CONSORT flow diagram.

Of the 53 patients assigned to the intervention, 27 were placed in the control group and 26 in the 3D‐replica group. A total of 46 patients completed the trial. Figure 2 presents the CONSORT flow diagram, illustrating the study participants' enrollment, allocation, follow‐up, and data analysis.

The 3D‐replica group (*N* = 24) comprised 54% female and 46% male patients, with a median age of 16 years (IQR: 16–17). The control group (*N* = 22) included 73% female and 27% male patients, with a median age of 17.5 years (Inter Quartile Range (IQR): 17–19). The total surgery time was shorter in the 3D‐replica group (45 min, IQR: 40–51) compared to the control group (60 min, IQR: 50–70), and extra‐alveolar time was shorter in the 3D‐replica group (35 s, IQR: 30–60) versus the control group (55 s, IQR: 30–83). Regarding donor fitting times, 63% of the 3D‐replica group required only one fitting, compared to 32% in the control group. The distribution of Moorrees' stages was similar between the groups, with most patients in stages 3 and 4 (Table 1).

**TABLE 1:** Demographic and clinical characteristics of the patients and surgical procedure.

Characteristic	3D Replica, *N* = 24^a^	Control, *N* = 22^a^
Sex		
Female	13 (54%)	16 (73%)
Male	11 (46%)	6 (27%)
Age (surgery)	16.00 (16.00, 17.00)	17.50 (17.00, 19.00)
Total surgery time (min)	45 (40, 51)	60 (50, 70)
Extra‐alveolar time (s)	35 (30, 60)	55 (30, 83)
Donor fitting times		
1	15 (63%)	7 (32%)
2	5 (21%)	8 (36%)
3	4 (17%)	5 (23%)
4	0 (0%)	1 (4.5%)
5	0 (0%)	1 (4.5%)
Moorrees' stage		
3	9 (38%)	8 (36%)
4	12 (50%)	6 (27%)
5	3 (13%)	8 (36%)

^a^ *n* (%); Median (IQR).

### 3.2 Clinical Outcomes

Both groups had 100% survival at 1 year. At 1 year, the percentages for each group were as follows: mobility 3D‐replica group 0%, control group 13.6%; bleeding—3D‐replica group 4.1%, control group 13.6%; and periodontal pockets—3D‐replica group 4.1%, control group 13.6%. Only adjusted multivariable analyses were performed because there were differences in baseline conditions between the groups. The linear mixed models fitted to predict mobility, bleeding, and pocket depth outcomes demonstrated substantial explanatory power, with conditional *R* ^2^ values indicating that the models explained a significant proportion of the outcomes (mobility, 51%; bleeding, 91%; pocket, 91%). After adjusting for other clinical variables, the treatment group (3D‐replica vs. control) did not significantly predict mobility, bleeding, or pocket depth outcomes. Instead, operative time, Moorrees' Stage, and specific donor fit times were key in explaining the differences in these outcomes. The details of each clinical outcome (Table 2 and Figure S2) are as follows:

1. Mobility: This model explained 45% of the outcomes. Mobility decreased significantly over time (6 months: *β* = −0.38, *p* < 0.001; 12 months: *β* = −0.42, *p* < 0.001). The treatment group did not significantly affect mobility (*β* = 0.12, *p* = 0.258). Significant predictors included total surgery time (*β* = 0.0043, *p* = 0.049) and Moorrees' Stage (Stage 4: *β* = −0.31, *p* < 0.001; Stage 5: *β* = −0.39, *p* < 0.001).
2. Bleeding: This model explained 48% of the outcomes. The bleeding outcomes remained stable over time (6 months: *β* = 0.04, *p* = 0.093; 12 months: *β* = 0.04, *p* = 0.093). The treatment group did not significantly affect bleeding (*β* = 0.07, *p* = 0.335). Significant predictors included total surgery time (*β* = 0.0049, *p* = 0.017), Moorrees' stage (Stage 4: *β* = −0.18, *p* = 0.022), and donor fitting times of 4 (*β* = 0.93, *p* < 0.001).
3. Pocket depth: The model explained 48% of the outcomes. The pocket depth did not change significantly over time (6 months: *β* = 0.04, *p* = 0.093; 12 months: *β* = 0.04, *p* = 0.093). The treatment group did not significantly affect the pocket depth (*β* = 0.07, *p* = 0.335). Significant predictors included total surgery time (*β* = 0.0049, *p* = 0.017), Moorrees' stage (Stage 4: *β* = −0.18, *p* = 0.022), and donor fitting times of 4 (*β* = 0.93, *p* < 0.001).

**TABLE 2:** Regression coefficients and statistical significance for predicting mobility, bleeding, and pocket depth in linear mixed models.

Characteristic	Mobility			Bleeding on probing			Pocket		
	*β*	95% CI	*p*	*β*	95% CI	*p*	*β*	95% CI	*p*
Group									
3D replica	—	—		—	—		—	—	
Control	0.12	−0.09, 0.33	0.3	0.07	−0.08, 0.23	0.3	0.07	−0.08, 0.23	0.3
Time									
3 m	—	—		—	—		—	—	
6 m	−0.38	−0.55, −0.20	< 0.001	0.04	−0.01, 0.09	0.094	0.04	−0.01, 0.09	0.094
12 m	−0.42	−0.59, −0.24	< 0.001	0.04	−0.01, 0.09	0.094	0.04	−0.01, 0.09	0.094
Age (surgery)	0	−0.04, 0.05	> 0.9	0.01	−0.04, 0.05	0.7	0.01	−0.04, 0.05	0.7
Sex									
Female	—	—		—	—		—	—	
Male	0.06	−0.09, 0.20	0.4	0.13	−0.01, 0.26	0.071	0.13	−0.01, 0.26	0.071
Total surgery time (min)	0	0.00, 0.01	0.055	0	0.00, 0.01	0.021	0	0.00, 0.01	0.021
Extra‐alveolar time (s)	0	0.00, 0.00	0.2	0	0.00, 0.00	0.9	0	0.00, 0.00	0.9
Moorrees' stage									
3	—	—		—	—		—	—	
4	−0.31	−0.47, −0.15	< 0.001	−0.18	−0.33, −0.02	0.027	−0.18	−0.33, −0.02	0.027
5	−0.39	−0.60, −0.18	< 0.001	−0.18	−0.38, 0.01	0.068	−0.18	−0.38, 0.01	0.068
Donor fitting times									
1	—	—		—	—		—	—	
2	−0.01	−0.18, 0.16	> 0.9	−0.14	−0.30, 0.02	0.083	−0.14	−0.30, 0.02	0.083
3	−0.05	−0.28, 0.18	0.6	−0.11	−0.33, 0.10	0.3	−0.11	−0.33, 0.10	0.3
4	1	0.43, 1.6	0.001	0.93	0.37, 1.5	0.002	0.93	0.37, 1.5	0.002
5	−0.25	−0.87, 0.37	0.4	−0.31	−0.89, 0.28	0.3	−0.31	−0.89, 0.28	0.3
Group * time									
Control * 6 m	0.01	−0.24, 0.27	> 0.9	−0.04	−0.11, 0.03	0.2	−0.04	−0.11, 0.03	0.2
Control * 12 m	−0.04	−0.29, 0.22	0.8	−0.04	−0.11, 0.03	0.2	−0.04	−0.11, 0.03	0.2

Abbreviation: CI, confidence interval.

### 3.3 Radiological Outcomes

The analysis of radiological outcomes revealed no statistically significant differences between the 3D‐replica and control groups across various measures (Table 3). For crown changes, the median crown change was 1 (IQR: −5, 3) in the control group and 1 (IQR: −4, 17) in the 3D‐replica group (*p* = 0.4, Wilcoxon rank sum test). Root changes showed a median of 19 (IQR: 3, 31) in the control group and 27 (IQR: 12, 36) in the 3D‐replica group (*p* = 0.15, Wilcoxon rank sum test). The distribution of periapical status was as follows: 50% normal in the control group compared to 43% in the 3D‐replica group; 35% with widening periodontal space/loss of lamina dura in the control group compared to 48% in the 3D‐replica group; and 15% with a radiolucent periapical lesion in the control group compared to 8.7% in the 3D‐replica group (*p* = 0.7, Fisher's exact test). Root development was 64% in the control group and 79% in the 3D‐replica group. No change was observed in 18% of both groups, and no development was observed in 18% of the control group and 4.2% of the 3D‐replica group (*p* = 0.3, Fisher's exact test). For external root resorption, 59% in the control group and 88% in the 3D‐replica group showed no resorption. In comparison, 36% and 13% of the control and 3D‐replica groups had resorption (*p* = 0.057, Fisher's exact test) (see Figure S1). The Modeling results further show a lack of significant differences. Table 4 presents the multivariate logistic regression analysis results, which evaluate the association between various radiological predictors and the likelihood of belonging to the 3D‐replica or Control group. The logistic regression model showed no significant associations for obliteration (*p* = 0.4), periapical status categories (*p* > 0.9, 0.5, and > 0.9, normal), widening periodontal space/loss of lamina dura, radiolucent periapical lesion, root development (*p* = 0.8 and 0.2 for no change and no development, respectively), or external root resorption (*p* = 0.087).

**TABLE 3:** Univariate analysis of radiological outcomes of control versus 3D replica group.

Characteristic	Control (*N* = 221)	Replica (*N* = 241)	*p*
Root development			0.3
Yes	14 (64%)	19 (79%)	
No change	4 (18%)	4 (17%)	
No	4 (18%)	1 (4.2%)	
Root resorption			0.057
No	13 (59%)	21 (88%)	
Yes	8 (36%)	3 (13%)	
Not available	1 (4.5%)	0 (0%)	
Periapical status			0.7
0 Normal	10 (50%)	10 (43%)	
1 Widening periodontal space/loss of lamina dura	7 (35%)	11 (48%)	
2 Radiolucent periapical lesion	3 (15%)	2 (8.7%)	
Crown/root ratio			0.4
Median (IQR)	1 (−5, 3)	1 (−4, 17)	

*Note: p* values for crown change and root change are from the Wilcoxon rank sum test; *p* values for periapical status, root development, and external root resorption are from Fisher's exact test.

**TABLE 4:** Multivariate logistic regression analysis of control versus 3D replica group.

Characteristic	log(OR)	95% CI	*p*
Root development (reference: Yes)			
No change	−0.34	−2.7, 1.9	0.8
No	−2.1	−6.3, 0.72	0.2
Root resorption (reference: No)			
Yes	−1.5	−3.4, 0.15	0.087
Not available	−17		> 0.9
Periapical status (reference: 0 Normal)			
1 Widening periodontal space/loss of lamina dura	0.55	−1.1, 2.3	0.5
2 Radiolucent periapical lesion	0.07	−4.0, 4.2	> 0.9
Crown ratio change (reference: No)			
Yes	−1.6	−5.6, 1.5	0.4

## 4 Discussion

In this controlled clinical trial, the use of 3D‐printed replicas and cone beam computed tomography (CBCT) as an adjunct to autotransplantation of molars showed no significant differences in clinical or radiographic outcomes compared to a control group that did not use this protocol. Both groups had high success rates, with no significant differences in outcomes attributable to 3D‐Replica use. Significant predictors of clinical outcomes included operative time, Moorrees stage, and donor placement time, but not group assignment.

A possible explanation for these findings in molars with open roots may be the inherently high success rates of autotransplantation, over 90% after 1 year at least [21, 22, 23, 24], which may overshadow the benefits of additional technologies such as CBCT and 3D‐printed replicas.

Previous observational studies have suggested differences in outcomes when using advanced imaging and 3D printing techniques for tooth autotransplantation [8]. However, these studies lacked controlled settings, and our study provides more robust evidence by eliminating the biases associated with observational data. In this regard, our results agree with those of another controlled clinical trial by EzEldeen et al. [7], which found no significant differences in clinical or radiographic outcomes between CBCT‐guided and conventional methods.

EzEldeen's study used a CBCT‐guided protocol that included a preoperative low‐dose CBCT scan, digital 3D modeling, virtual tooth autotransplantation planning, 3D‐printed tooth replica and surgical guide fabrication, and surgery under general anesthesia [7]. In addition, their study was limited to autotransplanted premolars. In contrast, we used standard‐dose CBCT, performed procedures without general anesthesia, and included molars in our study. Unlike EzEldeen, we considered the root development stage as a potential confounder, acknowledging its impact on healing and integration success rates. Therefore, our results extend the findings of Ez‐Eldeen et al. to multiradicular teeth and demonstrate that the lack of significant differences in outcomes holds across tooth types, developmental stages, and procedural variations.

Although these advanced techniques might not offer substantial clinical benefits in autotransplantation, they still hold value for specific scenarios. For instance, 3D‐replicas can enhance surgical precision, reduce extraoral time, and streamline procedures, which could be particularly beneficial in high‐volume surgical centers or for training novice surgeons [12]. Thus, the primary utility of these technologies may lie in optimizing surgical efficiency and providing valuable training tools for new practitioners rather than enhancing biological outcomes directly. This aligns with previous findings highlighting the efficacy of CBCT and 3D printing for accurate surgical planning and execution [7, 8, 12].

While CBCT offers advantages for surgical planning and precision in autotransplantation, its cost‐effectiveness and radiation dose must be carefully considered. CBCT is not as readily available as 2D imaging techniques like orthopantomography or retroalveolar radiographs, and it subjects patients to a higher radiation dose. For example, effective doses for children from CBCT range widely from 13 to 769 μSv, making age a crucial factor when assessing radiation exposure [25]. Overall, CBCT radiation exposure can be 15–140 times higher than 2D radiographic examinations [26], with an estimated cancer risk of 2.7–9.8 per million for a single scan [27]. Although the risk is considered low, careful justification, optimization, and clear communication of radiation risks to patients are essential for the responsible use of CBCT in clinical practice [28]. Also, studies in orthodontic planning show that CBCT does not significantly alter treatment decisions compared to 2D radiographs, though it increases examination costs [29]. Based on this, our findings suggest that 3D‐replicas should be reserved for complex cases requiring enhanced precision or for training purposes.

This study had limitations. A single surgeon performed all operations, which could enhance outcomes over time due to increased proficiency while minimizing inter‐operator variability. While the surgeon's skill could be considered a factor for autotransplantation success, evidence of its impact is mixed. Jakobsen et al. (2018) found no significant difference in outcomes between experienced and less experienced surgeons when assessing retrospectively 89 autotransplanted teeth [30]. However, Shetty and Ahmed suggested that the surgeon's skill in case selection and procedure execution is key to success [31]. This is consistent with a US study of 8503 experienced and 2119 new surgeons, which found that the majority of the difference in outcomes between new and experienced surgeons is related to the context in which care is delivered and the complexity of the patient, rather than the inexperience of the new surgeon [32]. In our study, we used a single experienced surgeon to ensure consistency in case selection and performance and to isolate the effect of the intervention on outcomes.

Additionally, our study was limited to a follow‐up period of 1 year. However, most studies have shown that complications after autotransplantation, such as ankylosis, root resorption, and pulp necrosis, occur within the first year [2, 5, 6, 13, 20, 33]. Long‐term complications are rare. For example, a systematic review and meta‐analysis on autotransplantation of teeth with incomplete root formation reported that the survival rates after 1, 5, and 10 years were 97.4%, 97.8%, and 96.3%, respectively, with annual complication rates for ankylosis at 2.0%, root resorption at 2.9%, and pulp necrosis at 3.3% [2, 5, 6]. These reports confirm that most complications tend to manifest within the first year after autotransplantation, supporting our decision to report after 1 year. While extending the follow‐up period could provide further insights, a 1‐year follow‐up offers a reliable snapshot of the procedure's success and associated complications. Furthermore, while our study focused on clinical and radiographic outcomes, future research could include other important outcomes such as patient satisfaction, quality of life [34], and cost‐benefit analysis. Investigating the specific conditions under which CBCT and 3D‐replicas offer clinical advantages could help refine their use in dental practice. For instance, exploring their application in cases of severe anatomical challenges or high‐risk patients could provide further insights.

In summary, while our results indicate no significant differences in outcomes between the CBCT/3D‐replica protocol and conventional methods after adjusting for other clinical factors, the potential for these technologies to enhance surgical efficiency and serve as valuable training tools warrants further investigation. Future studies should address these limitations and explore the broader implications of these advanced techniques in clinical practice.

## Author Contributions

**Miks Lejnieks:** conceptualization, investigation, methodology, project administration, validation, visualization, review and editing. **Ilze Akota:** conceptualization, review and editing. **Gundega Jākobsone:** review and editing. **Laura Neimane:** investigation, funding acquisition, review and editing. **Sergio E. Uribe:** investigation, data curation, formal analysis, investigation, methodology, visualization, validation, writing – original draft, writing – review and editing.

## Conflicts of Interest

The authors declare no conflicts of interest.

## Supporting information


Data S1.

## Data Availability

The data and analysis scripts supporting this study's results are available in Lejnieks and Uribe (2024). DATASET Clinical and Radiographic Evaluation Data of Autotransplantation using 3D‐replicas [Data set]. Zenodo. https://doi.org/10.5281/zenodo.13208598.
