# Supplementary material for: Clinical Efficacy of CBCT and 3D‐Printed Replicas in Molar Autotransplantation: A Controlled Clinical Trial
Source: Dent Traumatol. 2024 Nov 6;41(2):161–70. doi: 10.1111/edt.13012 (PMC11907221; doi:10.1111/edt.13012)
Supplement: Supplementary file 1 — Data S1. [file EDT-41-161-s001.zip › Supp Figures .docx]

**Supplementary Figure 1.** Clinical outcome changes over time between the 3D Replica and Control groups.

(A) Change in Mobility Over Time: Proportion of normal (yellow) and abnormal (purple) tooth mobility assessed at 3, 6, and 12 months post-transplantation. No significant differences were observed between the 3D Replica and Control groups at any time.

(B) Change in Bleeding on Probing Over Time: Proportion of normal (yellow) and abnormal (purple) bleeding on probing at 3, 6, and 12 months. Similar trends in both groups were noted, with most patients exhibiting normal values over time.

(C) Change in Periodontal Pocket Depth Over Time: Proportion of normal (yellow) and abnormal (purple) periodontal pocket depth at 3, 6, and 12 months. The 3D Replica and Control groups showed consistent clinical outcomes across the follow-up periods.

**Supplementary Figure 2.** Predicted values of clinical outcomes from linear mixed models.

(A) Predicted Values of Mobility: Predicted mobility values for the 3D Replica and Control groups at 3, 6, and 12 months post-transplantation. Mobility decreased significantly over time in both groups, but no significant differences were observed between the treatment groups (p = 0.258).

(B) Predicted Values of Bleeding on Probing: Predicted bleeding on probing outcomes for both groups across the same time points. Bleeding remained stable over time, with no significant difference between the groups (p = 0.335).

(C) Predicted Periodontal Pocket Depth Values: Predicted periodontal pocket depth values in the 3D Replica and Control groups. The pocket depth did not show significant changes over time or between groups (p = 0.335).
